# From embodiment to evidence: The harmful intersection of poor regulation of medical implants and obstructed narratives in embodied experiences of failed metal-on-metal hips

**DOI:** 10.1177/13634593231179026

**Published:** 2023-06-05

**Authors:** Pauline McCormack

**Affiliations:** Newcastle University, UK

**Keywords:** implant failure, medical device regulation, metal-on-metal hips, orthopaedic surgery, patient narratives

## Abstract

This research presents the results of a study about people with failed metal-on-metal hip implants, and draws on the STS concept of the technological imperative alongside research on the value of patient knowledge in clinical settings and the legitimacy of embodied stories. Popularly understood as positive and life changing, hip replacement surgery was hailed as ‘the operation of the century’, until a series of widespread failures of hundreds of thousands of hip implants, known collectively as metal-on-metal (MoM) hips, drew attention to the poor regulation of medical implants. This paper argues that poor regulation intersects with narratives of patients’ pain, which are obstructed by surgeons and the UK regulatory body, with the effect of denying both patients’ embodied experiences of implant failure, and their restitution to good health. Patient narratives about problems with their hip implant are the wellspring from which scientific evidence emerges which can indicate widespread implant failure. By obstructing these narratives the regulatory system undermines the very evidence it needs to operate effectively.

## Introduction

Within Science and Technology Studies (STS) there is a myriad of work which seeks to complicate biomedical approaches to innovations in healthcare technology, their regulation, and patient safety. It does so by highlighting the social dynamics embedded in the processes involved in innovation and regulation (Jasanoff, 2005). STS defines the biomedical approach as one that sees the governance of new technologies as driven by valuing newness and equating it to progress, and regulation as a safeguard that operates with a high level of scientific certainty (Brown and Webster, 2004). This way of framing is understood by STS as overly deterministic, unable to recognise the everyday practices which mediate the risks inherent in new technologies and their introduction into the human body (Allen et al., 2016; Foucault, 1973; Nettleton, 2020). This paper outlines how these tensions are present at the intersection of power relationships in healthcare, and health technology regulation – it contributes to a tradition of embodiment scholarship and shows negative healthcare experiences are exacerbated by ineffective regulatory systems. It presents the findings of a qualitative research study involving focus groups with 38 people with failed metal-on-metal (MoM) hip implants and their families, exploring their experiences of clinical encounters following failure as well as their views on implant regulation. It is also informed by participatory, action-research over several years with a patient advocacy group. The negative health consequences of a failed implant can be profound, and incidences of widespread failure are not isolated (Cumberlege, 2020; Wienroth et al., 2014). Social issues, and their specific influence on implant failures are largely neglected and it is important to understand the full range of contributory factors if future widescale failures are to be prevented.

It is widely acknowledged that the EU/UK regulatory system permits unsafe implants to be marketed (Cohen, 2011; Heneghan et al., 2011; Kent, 2003; Kramer et al., 2012). This study outlines newly identified gaps in the regulatory process around communication of implant failure to surgeons and patients and shows how these gaps intersect with the obstruction of patient narratives and an overreliance on technology, to derail and delay the reporting of failures. Patients in these cases experience ‘a sense of abandonment’ as well as ‘life changing consequences’ (Cumberlege, 2020: 4).

Metal-on-metal hip implants were developed in the late 1990s with later models in the early 2000s marketed as having a longer life in-vivo than existing implants, thereby making them desirable to patients and surgeons (Cohen, 2011). Problematic rates of failure were first highlighted in 2007 but it was 2010 before manufacturers began to withdraw products from sale. The effect of a failed implant can be life changing; patients in this study reported poor mobility and pain, leading to disrupted social and family lives, loss of livelihoods, curtailment of independence, and anxiety, distress and depression including one case of attempted suicide.

This work uniquely brings together theoretical perspectives from STS on how contemporary societies understand and enact technological regulation, with scholarship on the role of embodied narratives and how these are handled in clinical encounters for patients with unexplained symptoms (Frank, 2013; Fullagar and O’Brien, 2013; Manderson et al., 2008; Turner, 2008). This research’s new contribution to scholarship is in showing how patients’ accounts are undermined by regulatory dynamics and paradoxically, how the regulatory system is reliant on patient reports. Patients’ stories about problems with their hip implant are the wellspring from which evidence emerges – their recognition in formal health records and the accumulation of these can indicate widespread implant failure – and yet these stories are supressed by a combination of the denial of embodied realities and regulatory dysfunction (Jung, 2002; Jutel, 2019; van der Kamp et al., 2022; Verdonk et al., 2009).

This paper firstly discusses how patients presenting to their implant surgeons in pain or discomfort are judged to have an unexplained illness or symptoms of a physiological problem unrelated to the hip implant and, for women in particular, how their symptoms are often misinterpreted as psychosomatic.

Secondly, this work identifies how the positioning, by manufacturers, surgeons and the regulatory body, of scientific knowledge as certain, fixed and measurable alongside the valorisation of new healthcare technologies gives primacy to the performance of the implant. I discuss how the scientific knowledge, the imparting of such knowledge by the surgeon, and the stability of the implant, are viewed as inviolable and how this thwarts embodied knowledge and the patients’ agency.

Lastly, it is argued that the level of evidence about the performance of an implant required by the regulatory system is insubstantial and largely unverifiable and this not only allows faulty implants to come to market, but results in paltry evidence being available to orthopaedic surgeons. Additionally, the UK regulatory body does not effectively intervene when failures are identified.

## The role of technoscientific knowledge in patient’s unexplained pain

This paper draws from STS literature to outline three pillars of assumption which can be understood as being embedded in the introduction of new health technologies – assumptions which work to denigrate the patient experience. Firstly, new medical technologies, and data about them, are often considered scientific, authoritative and dependable by both health professionals and patients (Calnan, 1984; Frank, 2013; Lehoux et al., 2010; Salmon, 2000). In contrast, patient testimonies about a failing technology, such as implants, are often seen as anecdotal and lacking in evidence (Allen et al., 2016). As Calnan outlines, ‘medicine’s professional authority rests on its claims to have exclusive access to a superior body of knowledge and expertise’ (Calnan, 1984: 74), and this means that patients’ embodied knowledge is often side-lined (Berndt and Bell, 2021).

The second pillar is the technological imperative. Koenig (1988) used the term to characterise the actions of doctors who implement novel technologies, even when there is little evidence that they are safe or effective. The technological imperative is accompanied by the supposition that a new version or model of a technology will act in the same, predictable way as previous versions (Brown and Webster, 2004; Jasanoff and Kim, 2009; Martin et al., 2008).

The third pillar is professional expertise involving the mediation of the first two pillars through the expert assessment of the doctor or regulatory body. Patients receive their information about a new implant from their surgeon who they expect to be cognisant with regulatory information on safety. As Jasanoff writes, ‘experts are the people to whom publics turn for answers to the crucial regulating question, “How safe is safe enough?”’ (Jasanoff, 2005: 242). The surgeon also receives information in the other direction – from the patient. They are the first healthcare professionals, who are also part of the regulatory system, to hear the patients’ complaints about the implant causing pain or discomfort. As the recipients of patient narratives surgeons are mediators of this empirical evidence, and hold the power to decide if a patient’s story of a failing technology is counted or discounted (Asbring and Närvänen, 2004; Carel and Kidd, 2017; Toye et al., 2006).

In the case of MoM hips, the power of these three assumptions is bolstered by the successful track record of hip replacement procedures in general, and their place in the popular and clinical imagination as a positive, life-changing technology (Learmonth et al., 2007). The use of a new model of hip is partly premised on this, and its introduction benefits from its impressive reputation.

These pillars create the expectation that each technological adaptation will act in the same way as previous versions of the technology, in a positive and foreseeable manner (Borup et al., 2006). The consequence is that when patients report failures they are offering a story which does not fit this expectation and which the surgeon can find hard to accept (Carel and Kidd, 2017; Fullagar and O’Brien, 2013). The stories people tell about illness are crucial in uncovering their understanding of their diseased body and in transferring and negotiating that understanding with others. ‘These embodied stories have two sides, one personal and the other social’ (Frank, 2013: p2), and included in the social is the telling of the story to someone else. Frank writes of several narrative types including the Restitution Narrative where the ill person seeks medical intervention in pursuit of a return to ‘normality’. This quest for normality is hampered when narratives of pain and unexplained symptoms are not taken seriously in the clinical setting (Antelius, 2009; Foss and Sundby, 2003; Nettleton, 2006) and patients’ stories are not seen as credible or objective (Grace and MacBride-Stewart, 2007; Nettleton et al., 2004; Richardson, 2005; Toye et al., 2006).

The patient is then forced into what Frank terms, a Chaos Narrative, the opposite of restitution, which is characterised by uncertainty, structurelessness and a difficulty in being heard (Frank, 2013). Lillrank (2003) asserts that this can be because symptoms which cannot be explained challenge, ‘the central biomedical epistemology; objective knowledge and measurable findings’ (Lillrank, 2003: 1049). This can be complicated by the social and political expectations of the surgeon’s role as holder of certainty, which may hamper their speaking out about fears around patient safety (Allen et al., 2016; Berman and Ogden, 2017). Voicing these concerns can be disruptive to the professional setting and to professional identify (Keating and Cambrosio, 2003) and is, ‘highly context-dependent and embedded in the daily interaction rituals that suffuse work in a complex organisation’ (Szymczak, 2016).

This is all particularly acute for women, where well-established debates connect structural inequalities to women’s accounts of pain and unexplained symptoms, showing that these accounts are less likely to be taken seriously in the clinical setting (Doyal, 1995; Foss and Sundby, 2003). In this context it is argued that women are not seen as credible or objective (Grace and MacBride-Stewart, 2007; Nettleton et al., 2004; Richardson, 2005; Toye et al., 2006) and as such doctors are more likely to assign a psychological or emotional reason for women’s symptoms, than a physical one (Kent, 2003; Lillrank, 2003; Verdonk et al., 2009), using phrases such as, ‘“it is only in your head”’ (Moss and Dyck, 2003: 89). A further aggravating feature is that women having hip implants tend to be older, and older women are seen as fragile and passive in the medical encounter, meaning narratives which contradict the clinician are unlikely to be countenanced (Reventlow et al., 2006).

Patient’s stories of implant failure therefore dispute all three pillars of assumption outlined above by signalling that the new technology, along with the clinicians’ and regulatory body’s assessment of it, are unreliable.

## The problem with implant regulation

To understand how patients’ narratives about their problems with MoM have been handled, it is also important to place this research within the context of the regulation of implants. Numerous problems are indicated in the literature and this paper concentrates on two aspects which are most relevant to this study, namely, lack of pre-market scrutiny of implants and an absence of post-market leadership from the regulatory body, the Medicines and Healthcare products Regulatory Agency (MHRA).^1^

The first major problem is that medical implants are subject to far less scrutiny than drugs and approval for a new implant is granted by a private company called a Notified Body which is commissioned and paid by the implant manufacturer. The evidence provided to the Notified Body is compiled by the manufacturer with no requirement for it to be publicly available, and evidence is presented to surgeons by the manufacturer’s sales representatives who have a financial interest in the implant being accepted for use (Blume, 2013; Schott et al., 2010; Stephens et al., 2006). Seemingly established implants are subject to even less scrutiny because the manufacturer can claim ‘substantial equivalence’, saying that the new implant is similar to an existing one with only minor changes to the design. Here the standard of evidence required is very low, the approval is fast-tracked, and there seems to be no recognition that a small change in implant design can result in a big difference in performance (The Royal College of Surgeons of England, 2001). Implants therefore become available with very little accompanying data about performance, none of it independently produced or scrutinised (Cohen, 2012a; Faulkner and Kent, 2001). As one epidemiologist put it, ‘We shouldn’t be in this position where we don’t know’ (Cohen, 2012b: 19). This special track for ‘established’ implants is one of three layers of presumption about the certainty of the implant and its performance, the other two being the technological imperative and the popular view of hip implants, in particular, as *the* medical success story (Learmonth et al., 2007).

The second problem is that, when implant failures are indicated, the UK regulator, the MHRA, operates reactively and often too late to prevent failures from becoming widespread. The BMJ outlines how harm was caused to large numbers of patients when regulators and manufacturers knew about the risks of MoM hips and did not communicate this risk (BMJ, 2012). Data showing large scale failures first emerged via registries (which collect data on the number of patients whose first hip implant is replaced), firstly in the Australian Joint Registry and later in the UK National Joint Registry (NJR) and although the MHRA appointed a committee to review the data, they did not issue any guidance for another 3 years (Tucker et al., 2011). During this time surgeries using MoM hips peaked, with thousands being newly implanted in the UK. Commenting on the MHRA’s approach to implant regulation the Lancet concluded, ‘these serious examples of device failures result from MHRA’s paralysis and inability to address the shortcomings of a badly flawed system’ (The Lancet, 2012: 2402) and ‘The operating principle at the MHRA seems to be do nothing until something goes wrong’ (Horton, 2012: 106).

There is a critical interplay between the comparative lack of scrutiny of implants by regulators, pre- and post-market, and patients not being believed by surgeons. In the absence of oversight the NJR is the only site where reliable, publicly available data showing patterns of failure in-vivo, is collected. Data only appears in the NJR when a surgeon decides that an implant has failed and needs to be replaced, and to do this the surgeon needs to have listened to and investigated patient narratives of pain and discomfort. These testimonies only transmute into evidence of implant failure if they are taken seriously and believed.

This paper investigates this interplay through a study undertaken with a group of people with failed MoM hip implants based in the north of England which explored their lived experience of implant failure and their views on implant regulation.

### When technology fails patients

When metal-on-metal hip failures began emerging as a widespread problem, little was known about the lived experience of people with failed implants or the impact on their lives. This project was funded by a Newcastle University EPSRC Impact Grant entitled, ‘When technology fails patients. Engaging with stakeholders about the case of the ASR^2^ hip joint failures’ (PI Prof T J Joyce). The engagement enabled repeated conversations with patients, most of whom thought their voices were not being heard and that their perspective was important in helping to understand problems with failed hip implants. The research presented in this study was opportunistic and was undertaken to explore two areas: (i) individuals’ daily experiences, their health problems, and the impact of the failure on their lives and (ii) the opinions and views of people with failed implants and their families on wider policy concerns and on the actions of relevant authorities on the issues.

This article reports on the latter, having gathered data in focus groups involving 38 people, 30 of them had at least one failed MoM hip implant, one had two functioning MoM implants and there were seven accompanying family members/friends. About 67% (20) of the failed implant participants were women. It is recognised that the impacts of health problems are familial and it was therefore thought important to give the participants the opportunity to include a family member or friend.

Initial scoping, carried out with active volunteers from a patient support group in the north of England, Altogether Hip, identified questions repeatedly asked by patients. Firstly, which organisation(s) was responsible for managing the response to widespread failure? Secondly, what steps were taken to arrest, manage and remedy the situation? The focus groups aimed to explore patients’ and their families’ opinions of the systemic picture around the emergence of failures, involving such issues as healthcare, regulation of medical implants and the responsibility and limits of health professionals and industry. The following questions were put to participants for discussion:

- Who do you think is responsible for ‘what has happened’ with MoM hips?
- Who should be responsible for making sure the issues you have just discussed don’t happen again?
- Tell us how this has affected your trust in medicine.

Ethical approval was received from Newcastle University. Most participants were recruited through Altogether Hip, others were individuals who had independently contacted the research team, and all were volunteers. It was important to talk to people who were interested in the policy areas being explored, which is why most participants were members of a support group. The limiting factors of this method are that, because the group was self-selecting, it was possible that some heterogeneous views were not heard. Plus, geographical concentration meant a number of participants were treated at the same hospital and this has been taken into account when presenting the data.

The 38 participants were split into three groups of 12 or 13 people, each of which had a moderator. These groups were further subdivided into sets of 3–4 people to allow participants to work through the focus group questions. The 12/13 person groups were then reformed with the moderator to discuss their responses and these discussions were audio-recorded and transcribed verbatim. Reflective field notes were made following the focus groups which recorded information not discernible on the audio-recordings such as non-verbal communications and participant mood.

Moderators met later in a two sessions to discuss the outcomes of the focus groups and to inductively identify broad themes and tacit findings arising from the data which were mostly around problems with regulation. The moderators’ field notes were included in considerations during this meeting, the results of which were written up and agreed. The transcriptions were then analysed inductively by myself, using thematic analysis (Boyatzis, 1998) allowing concepts to emerge from patterns in the discussions. Thematic analysis lends itself to ‘similarities and differences and generating unanticipated insights’ (Nowell et al., 2017: 2) which made it appropriate for this study. Indeed, during detailed analysis an unanticipated theme highlighting the gendered nature of clinical interactions emerged and could be seen as interwoven with data about problems with regulation. A coding framework was established which allowed comparison and refinement of themes during multiple passes of the data until saturation had been reached and it was felt that the most significant social processes had been identified. Regular reflexive meetings with a critical peer took place during analysis, to discuss data and emerging conclusions in order to test the reliability of findings.

### Narratives of implant failure

As the patients embarked on a diagnostic odyssey, seeking a restitution narrative, aspects within the regulatory landscape and clinical encounters worked in concert to build barriers which obstructed the path to resolution.

In April 2010 a Medical Device Alert (MDA) recommending surveillance of MoM hip patients was issued by the MHRA (MHRA, 2010). This was the first time the regulatory body had indicated that all MoM patients were potentially at risk, in a communique issued to every healthcare trust in the UK for dissemination to ‘all relevant staff’ (ibid). The MDA stated that MoM patients should be recalled to their treating hospital and those with pain should have blood tests to check for levels of cobalt and chromium (recognised clinical markers of a failing hip implant) in the body.

A variety of scenarios were related by focus group participants about the recall. Some were simply not contacted by their treating hospital and were unaware they should be subject to surveillance – around a third of focus group participants claimed not to have been recalled and to have found out about it through other means.

Mandy: ‘*I read a short article in* [a magazine] *which said that there was a problem with some metal on metal hips . . .* [then]. . . *I met a nurse I knew who’d had a hip replacement herself and asked her advice. It was two years since her op and she had just had a recall letter from Hospital A. She suggested that I contact my surgeon. I hadn’t had a letter about the recall, I still haven’t had one to this day’*.

Suzanne: ‘*But everybody who had this. . .had these implants. . . .didn’t they get the* [recall] *letter?’*

Multiple voices (indignant): ‘*No!*’

Jill: ‘*I never got a letter*’.

Other patients discovered by chance that there were known problems with MoM hips and then went on to seek out information for themselves, often using the resources of the support group.

Lizzie: ‘*At one of the hip* [support group] *meetings we have got people coming from all over because there is nothing in their areas and a gentleman came from K area* [a city over 100 miles away] *and it wasn’t even acknowledged over there that there was any problem and he was coming over here to find out information to go back and tell his surgeon*’.

This last quote signals a critical position whereby some patients, particularly those involved in the support group, appear to be in possession of more up-to-date or more accurate knowledge than their surgeons. This points to a newly identified gap in the regulatory system whereby the regulatory body has no way of knowing if information about failed implants and required surveillance is reaching patients and even surgeons. UK regulation has been described as a ‘tenuous network’ (Turner, 2008; Wienroth et al., 2014: 12) and here we have evidence of a network which is perilously incomplete.

These failures of the regulatory system are amplified when patients have unsatisfactory encounters with surgeons and may be more noticeable for women, whose epistemic position is often questioned due to gendered practices. Several studies outline how men and women have different experiences when consulting with surgeons, meaning women wait longer for surgery and have a higher disease burden (Doyal, 1995; Hutchison, 2019; Toye et al., 2006). The majority of women in the focus groups said that their suspicions that pain and other symptoms were caused by a faulty hip implant were dismissed or downplayed by surgeons.

Edwina: ‘*One [surgeon] told me that I was wasting his time and there was nothing wrong with my hip and the pain was coming from my back’’*.

Lesley: ‘*What annoys me is why . . .why was I fobbed off? They kept saying “oh it’s a ligament problem” cos I had a pain in me groin, directly after me operation . . . and they just kept saying “oh it’s ligament, oh it’s ligament”*’.

Lisa: ‘*Yeah, I was gonna say that the consultant, especially the one I had was arrogant, fobbed me off, fobbed me off for two years, at one point he told me that I needed a holiday in a hot country’.*

Focus group members noted how attribution of women’s symptoms as imagined or psychological was in contrast to how men were treated during clinical consultations.

Cath: ‘*I’ve heard so many women say they’ve gone in and in terrible pain and everything and they’ve been just dismissed. “Oh it’s in your head, there’s nothing wrong with these hips”, like almost “go away dear”, you know, “go away dear, don’t be silly”. I’m sure to you men at the end they would be quite, but you know, certainly it’s usually male consultants and there is a bit of that attitude around*’.

None of the men in the focus groups reported such encounters and in response to this discussion one male participant noted:

Philip: ‘*I think there’s a general impression, from me certainly, and it is a generalisation about surgeons, they’re arrogant, . . . superior, all those sort of words, that they know best, you know like you said, “don’t worry dear it’ll be all right” cos they know best*’.

Here we see the obstruction of patients’ narratives of pain by surgeons, which is exacerbated by the technological imperative and by surgeons’ assumption that the performance of the hip implant is predictable and outcomes of the surgery will be as positive as they have previously been. Patient assertions that symptoms were caused by implant failure are therefore rendered illegitimate through the privileging of the surgeon’s assessment using scientific information, over embodied experience. Focus group participants believe that women meet with this behaviour more often, and this would fit well understood tropes.

These inaccurate responses by the surgeon has a number of consequences. Firstly, patient efforts to pursue a diagnosis and move towards restitution is suspended, meaning their hip damage worsens and they suffer for longer. Secondly, while the assertions that symptoms are due to a failing hip implant are deemed to be invalid, the collection of evidence that would indicate that failures are widespread, is delayed (Manderson et al., 2008). The patients are doubly disadvantaged as surgeons fail to vindicate patient accounts which seek a medical explanation, and also bring into question the veracity of patient testimonies.

An additional confounding factor about the hip implant’s performance is that clinicians’ knowledge could only ever be partial due to the lack of pre-market performance data which ensures the surgeon does not have access to independent evidence from clinical trials or testing. This presents a barrier to clinicians when they decide whether or not to give the patient testimonies epistemic worth, because such testimonies contradict the clinician’s sphere of knowledge. A sphere of knowledge which is undermined by the fact that the data which forms part of the clinicians’ decision-making is restricted by the inadequacy of the regulatory system and is insufficient to signal that the implant is problematic and failing.

Several people in our focus group talked of problems with the communication of blood test results as they sought to use the results as proof that their health problems were being caused by a failing implant.

Bernadette: ‘*Hospital D refused to give me my blood test results until I’d seen the surgeon. So I thought, I’ll go to my own GP and I got them and when I went to Hospital D, he said “how have you got hold of those?”, he was not pleased*’.

David: *‘Well my blood level^3^ was for instance like, my surgeon had, he sent a letter and he [his GP] read the letter in front of me and I didn’t look at it cos he had it on his computer screen and he said er, “he’s put down here your levels are okay” and so he believed, my GP believed the surgeon. I believed my GP, it was only when I got access to the details I said to my GP, “well, they’re not okay”. Right and he said, “well the surgeon said they’re okay” and I said, “I don’t care what he ses”’.*

Andrew: *[talking to surgeon] ‘I said anyway, how were my metal things (see Note 3) . . .“perfectly normal”. He didn’t give me them, he didn’t tell me what they were. I had a letter sent to my doctors and had to get a copy of that letter so I am reading it and I am thinking, I had better go back . . . and I listened to the [TV] programme^4^ about what the levels should have been and mine were like thirty times higher than this lot and I am thinking. . .he has lied to me then, again’.*

These exchanges are evidence of patient experiences of negative interactions with surgeons, and of surgeons’ displeasure with the patient’s lack of compliance, as they independently seek out information and clarification. The patient expects the surgeon to apply their professional expertise and to mediate scientific information about the implant and in these cases we see that mediation failing, with surgeons whose interpretation of the evidence is incorrect or out of date.

These negative interactions create tension as patients realise that the surgeon is not fulfilling their role as expert. Kent tells us of patients with faulty breast implants who, ‘developed their own “expertise” and drew on a stock of knowledge that enabled them to make sense of their lived experience and to challenge the “experts”’ (Kent, 2003: 417). The data above show people with faulty MoM implants doing them same, however, when they use their own knowledge and new expertise to confirm their embodied experience, it is challenged. As Bordo explains, ‘the medical model has a deep professional, economic and philosophical stake in preserving the integrity of what it has demarcated as its domain’, and arguments against this model are, ‘resisted more consciously and deliberately’ (Bordo, 2004: 53).

Participants in the focus groups recognised the value of their knowledge and embodied experiences in contributing to the discovery that there was widespread failure of MoM hip implants in that, their experiences had significance above and beyond the individual patient.

Carmel: *‘It was patients feeding back to him* [the surgeon] *– he should have taken notice of that [other voices: yep] he should have listened to their concerns. You don’t carry on doing things if the patients are showing that is*, *you know, going wrong’*.

Surgeons are the direct link between the patient and the regulatory system, are key arbiters of the patients’ knowledge, and the patients are totally dependent on the co-operation and facilitation of their surgeon. When surgeons believe patients, they can set in motion a process where: tests are undertaken; if necessary the faulty implant is replaced and at that point; details of the faulty implant are sent to the NJR. This is the place where evidence is compiled, analysed and reported on, with higher than expected failure rates being highlighted in the NJR’s Annual Report. It was only when surgeons began to use their professional expertise to facilitate information in the opposite direction – from patients to the regulatory system – that patient narratives began to constitute evidence.

Surgeons however, are only part of the tenuous network that makes up the regulatory system in the UK and conscious and deliberate resistance against challenges to the assumption of scientific certainty around MoM implants was also exercised by the regulatory body, the MHRA.

In a BBC investigation the MHRA’s then Chief Executive dismissed claims that more efficient regulation or earlier action by the MHRA might have prevented patient suffering and ill health, while the then Clinical Director of the MHRA played down the problems (BBC, 2012; Newsnight, 2012). This ultimately shows patient narratives being obstructed by the regulatory body, which continued to perpetuate a story where the scientific information about the implant was more reliable than patient evidence. Focus Group participants recognised this potential ‘sliding doors’ moment:

Kathleen: *‘And we felt very strongly that when you go to have such a big thing happen to you, you should be confident that the regulatory body has made sure that everything is correct, when you go to have this op there shouldn’t be any problem with these things that’ve been manufactured and I think we all felt that if someone had listened then results might have happened and everybody might not have had all these experiences to share*’.

Participants also criticised the MHRA for the perpetuation of weak, pre-market authorisation and for not using the powers it holds to issue warnings or to call for moratoria on medical implants.

Ann: *‘We feel that the regulatory bodies in this country have let us down badly.* *That is what they’re meant to be there for, to ensure that we have something that is* *there, that will be good for us and it will increase our level of health and instead we’ve had a product which really hasn’t been tested well and then when alarm bells are being rung, then they really haven’t necessarily acted quickly enough about it’.*

Cath: *‘The actual regulatory body, we were all really annoyed at that, . . . were really annoyed that the regulatory body, we felt that they shirked their responsibility and, what is the regulatory body? Has it not got enough teeth? We thought that there should be somebody looking at the regulatory body and there should be policing of the regulatory body, by a patient group or there should be somebody looking in. I mean, how they, they let this happen?’*

When it comes to the central premise of this article, that patient narratives constitute crucial evidence, the MHRA contributes to the derailing of these narratives and places additional obstructions on the patients’ paths to resitution, by denying there is a systemic problem with MoM hip implants (Langton et al., 2011; MHRA, 2012a, 2012b). The regulatory body’s continued minimising of problems further silences patients and slows down the collection of patient stories and their transformation into evidence. The turnaround with failed MoM hips began when surgeons, starting with a vanguard, began to listen to, and act on, patient concerns. Some of this turnaround was driven by patients who, as a result of negative interactions with their original surgeon, went ‘doctor shopping’, (Manderson et al., 2008: 523) seeking a clinician with a more sympathetic attitude. Our focus group participants swapped information with each other and many of them consulted one particular surgeon in the region who was known to take their concerns seriously.

One woman describes the relief when her embodied experience of pain was acknowledged:

Helen: *‘I ended up going for a* second *opinion and he patted me on my arm and said, “I am sorry you have been treated very badly” . . . You know your body yourself, you know when you are in pain’*.

Finding such a surgeon represented a reversal in treatment and a turnaround in how the clinical appointment was socially mediated, with dual, positive consequences of medical resolution for the patients and the contribution of evidence to the NJR. For these to happen a surgeon has to suspend belief in the technological imperative and entertain the possibility that patients’ embodied narratives are authentic, have validity and are worth exploring.

## Discussion

This research demonstrates how some surgeon’s unwavering adherence to techno-scientific certainty over embodied testimonies of ‘unexplained’ pain intersect with a malfunctioning UK (and EU) regulatory system to obstruct the identification of instances of systemic implant failure. Findings show how patients’ embodied knowledge about hip implant failure and its significance are at first dismissed, then derailed, before finally being transmuted into legitimate scientific evidence, which then becomes an influential force on the regulatory system. Unequal power relationships in healthcare and poor pre-market regulation are not new, but here we see the interaction of those two factors, and their interplay reinforcing each other and causing harm. There are several innovative scholarly contributions.

Firstly, this work demonstrates that patients’ embodied narratives are key to understanding the performance of medical implants and this needs to be more explicitly recognised by those working in the regulatory system, for outcomes with failed implants to improve. Surgeons are the direct, expert, mediators of evidence from the regulatory system to patients and vice-versa and therefore need to be aware of the possibility both of bias towards testimonies of unknown symptoms, and the limitations of evidence from pre-market authorisation. Poor pre-market regulation is normally understood to act in a material way, in permitting the authorisation of potentially faulty implants which can cause physical harm. We can now understand that it also acts in a social way by providing an inaccurate evidence base which influences surgeons’ reactions to, and conversations with, patients about a failing implant, leading to negative clinical interactions. Despite the evidence bases around many implants being inadequate, they exert a powerful social effect which can drive the contradiction or rejection of embodied evidence. In its widest sense, the lack of scrutiny of new implants shores up the technical imperative and the assumption that the implant cannot be at fault as there is no available evidence to the contrary.

Secondly, this work shows that there is a fissure when it comes to knowing whether or not patients are properly informed about implant failures, and possible surveillance or testing needed to identify failures. Effectively communicating such messages to patients should be considered part of the regulatory system, not simply a mundane function of healthcare and there is an obvious and serious gap in the regulatory system with regards to this. The MHRA is unaware of chaotic communication because, once they have written to hospitals, their involvement in communicating failures to patients ends. This is linked to the minimisation of implant failures which the MHRA practices which is a consequence of not giving sufficient consideration to patient concerns. At the moment the regulatory system is reliant on hospitals to contact patients and there is no feedback loop if hospitals do not complete this contact. The MHRA should take responsibility for knowing whether information on failures is being properly communicated to patients and institute a checking mechanism or feedback loop whereby hospitals or primary care practitioners confirm that at risk patients have been informed.

Thirdly, the actions of the MHRA contribute to delays in patient stories becoming evidence, by consistently and insistently denying there are widespread problems with implants. Patients expect the regulatory body to be concerned with their safety and to warn them in cases of potential harm. The regulatory body meanwhile puts forward an alternative account, that everything is (probably) okay. This approach has social heft as it offers reassurance to patients that they are probably unaffected – reassurance which is unwarranted. The regulatory body should take a more visible and explicit leadership role in cases of implant failure and adopt a precautionary or cautionary stance. Precaution would mean calling for a pause in the use of certain implants until their performance becomes clearer through the gathering and analysis of data, and caution would involve imposing a moratoria on the use of problematic implants. Either of these would have the dual purpose of (i) explicitly releasing information which informs patients and clinicians about potential risk and (ii) the countenancing of patient narratives, and which would prevent the regulatory system from obstructing the very evidence it needs to operate effectively.

## Conclusions

The findings in this study illuminate how patient accounts are crucial if regulatory management of implant failure is to be improved. It demonstrates systemic practice of obstructing patient narratives and an over-reliance on the infallibility of new technologies and the scientific information about them. These attitudes intersect with the deficient regulatory system and the problems exacerbate each other, so that the time taken for patient accounts of embodied experience to be transformed into scientific fact, is delayed. To constitute evidence stories have to accumulate – they are no good in isolation. The regulatory system is both supportive of, and dependent on, the assumed incontrovertibility of technoscientific knowledge and expertise, to the extent that the system is reluctant to allow alternative narratives or embodied knowledge from patients to be considered. Patients are not believed partly because surgeons and regulators, relying on existing scientific information, perceive there is insufficient evidence of implant failures, and there is insufficient evidence of implant failures because patients are not believed.

## Notes

1. This work makes an important distinction between the regulatory body (MHRA), which has an oversight function for all implant regulation and the regulatory system, which includes the MHRA as well as joint registries, surgeons, surgeons’ professional bodies, and manufacturers among others.

2. A model of MoM hip which was the first to show high failure rates.

3. Results of blood tests showing cobalt and chromium levels.

4. A 2012 episode of Newsnight which explored MoM hip failures (http://news.bbc.co.uk/1/hi/programmes/newsnight/9700909.stm).
